# Importance and challenges of measuring intrinsic foot muscle strength

**DOI:** 10.1186/1757-1146-5-29

**Published:** 2012-11-26

**Authors:** Achini Soysa, Claire Hiller, Kathryn Refshauge, Joshua Burns

**Affiliations:** 1 Arthritis & Musculoskeletal Research Group, Faculty of Health Science, University of Sydney, Sydney, Australia; 2 Institute for Neuroscience and Muscle Research/Paediatric Gait Analysis Service of NSW, Sydney Children’s Hospitals Network (Randwick and Westmead), Sydney, Australia

**Keywords:** Foot, Muscles, Toes, Muscle strength, Dynamometer

## Abstract

**Background:**

Intrinsic foot muscle weakness has been implicated in a range of foot deformities and disorders. However, to establish a relationship between intrinsic muscle weakness and foot pathology, an objective measure of intrinsic muscle strength is needed. The aim of this review was to provide an overview of the anatomy and role of intrinsic foot muscles, implications of intrinsic weakness and evaluate the different methods used to measure intrinsic foot muscle strength.

**Method:**

Literature was sourced from database searches of MEDLINE, PubMed, SCOPUS, Cochrane Library, PEDro and CINAHL up to June 2012.

**Results:**

There is no widely accepted method of measuring intrinsic foot muscle strength. Methods to estimate toe flexor muscle strength include the paper grip test, plantar pressure, toe dynamometry, and the intrinsic positive test. Hand-held dynamometry has excellent interrater and intrarater reliability and limits toe curling, which is an action hypothesised to activate extrinsic toe flexor muscles. However, it is unclear whether any method can actually isolate intrinsic muscle strength. Also most methods measure only toe flexor strength and other actions such as toe extension and abduction have not been adequately assessed. Indirect methods to investigate intrinsic muscle structure and performance include CT, ultrasonography, MRI, EMG, and muscle biopsy. Indirect methods often discriminate between intrinsic and extrinsic muscles, but lack the ability to measure muscle force.

**Conclusions:**

There are many challenges to accurately measure intrinsic muscle strength in isolation. Most studies have measured toe flexor strength as a surrogate measure of intrinsic muscle strength. Hand-held dynamometry appears to be a promising method of estimating intrinsic muscle strength. However, the contribution of extrinsic muscles cannot be excluded from toe flexor strength measurement. Future research should clarify the relative contribution of intrinsic and extrinsic muscles during intrinsic foot muscle strength testing.

## Introduction

Intrinsic foot muscles contribute to the support of the medial longitudinal arch [1,2] and are thought to work in conjunction with the plantar aponeurosis, plantar ligaments and extrinsic foot muscles to control the stresses on the foot during gait [3-5]. Intrinsic foot muscle weakness has also been implicated in the development of pes cavus in Charcot-Marie-Tooth disease (CMT) [6,7], heel pain [1,8,9], claw toe deformity [10], hammer toe deformity [10,11], and hallux valgus [10,12,13]. The level of intrinsic muscle weakness necessary for the development of these deformities and disorders is unknown. To assess the degree of weakness and to determine the effect of strengthening intrinsic muscles, a valid and reliable measure of intrinsic muscle strength is needed. There are diverse methods available for measuring intrinsic muscle properties [3,12-24], but there is lack of agreement regarding the most appropriate measure of strength. Therefore, the aim of this review was to provide an overview of the anatomy and role of intrinsic foot muscles, implications of intrinsic weakness and evaluate the different methods used to measure intrinsic foot muscle strength.

## Method

The electronic databases MEDLINE, PubMed, SCOPUS, Cochrane Library and CINAHL were searched between 21 May and 21 June 2012 to locate scientific articles on intrinsic foot muscles and muscle strength measurement. The main search terms and number of articles retrieved are listed in Table 1, 2, 3, 4 and 5. The search engine PEDro was also accessed and one article [25] was retrieved in the search results. Further articles were identified by hand searching reference lists of the extracted articles. Google Scholar was also searched to identify any relevant unpublished or in press articles using the same search terms as those used in the database searches. The abstracts of the located articles were then read to select the appropriate articles, with full copies of the articles examined if the study was relevant to the research aim.

**Table 1:** Database search strategy for PubMed

**Number**	**Search terms**	Results*
1.	foot	95709
2.	muscle	813998
3.	intrinsic	96812
4.	Number 1+2+3	260
5.	measure	487905
6.	strength	166587
7.	Number 1+2+3+5	18
8.	Number 1+2+3+6	28

*The results are from the search conducted on the electronic database PubMed between May and June 2012.

**Table 2:** Database search strategy for MEDLINE

**Number**	**Search terms**	Results*
1.	foot	43149
2.	muscle	297428
3.	intrinsic	59556
4.	Number 1+2+3	113
5.	measure	21182
6.	strength	110271
7.	Number 1+2+3+5	3
8.	Number 1+2+3+6	18

*The results are from the search conducted on the electronic database MEDLINE between May and June 2012.

**Table 3:** Database search strategy for EBSCO/CINAHL

**Number**	**Search terms**	Results*
1.	foot	21242
2.	muscle	55954
3.	intrinsic	4084
4.	Number 1+2+3	44
5.	measure	52005
6.	strength	28651
7.	Number 1+2+3+5	2
8.	Number 1+2+3+6	12

*The results are from the search conducted on the electronic database EBSCO/CINAHL between May and June 2012.

**Table 4:** Database search strategy for SCOPUS

**Number**	**Search terms**	Results*
1.	foot	161105
2.	muscle	1157744
3.	intrinsic	239220
4.	Number 1+2+3	349
5.	Measure	1433899
6.	Strength	891550
7.	Number 1+2+3+5	21
8.	Number 1+2+3+6	44

*The results are from the search conducted on the electronic database SCOPUS between May and June 2012.

**Table 5:** Database search strategy for Cochrane Library

**Number**	**Search terms**	Results*
1.	foot	4650
2.	muscle	23527
3.	intrinsic	1436
4.	Number 1+2+3	3

*The results are from the search conducted on the electronic database Cochrane Library between May and June 2012.

Fifty three research articles were identified that related to intrinsic foot muscles and strength measurement. Articles had to meet certain criteria for inclusion. The inclusion criteria were as follows.

(i) Research related to the role of intrinsic foot muscles

(ii) Research related to the anatomy of intrinsic foot muscles

(iii) Research describing the measurement of intrinsic muscles and toe muscle strength or weakness. Papers relating to the intrinsic foot muscle strength were considered initially, but it became apparent that few papers existed. Therefore the search was broadened to include articles relating to the measurement of toe muscles

(iv) Publication in peer-reviewed journals

(v) Full-text English language articles

### Anatomy of the intrinsic foot muscles

The plantar and dorsal intrinsic muscles of the foot have both their origin and insertion within the foot [26,27]. Intrinsic foot muscles differ from extrinsic foot muscles, which have their origins in the leg and the long tendons cross the ankle joint complex [27]. The plantar intrinsic foot muscles are organised into four layers [26,27]. The most superficial layer is deep to the *plantar aponeurosis* and includes the *abductor hallucis, flexor digitorum brevis*, and the *abductor digiti minimi*[26]. The second layer consists of the *quadratus plantae* and *the lumbricals*. The third layer consists of *adductor hallucis transverse*, *adductor hallucis oblique*, *flexor hallucis brevis* and *flexor digiti minimi brevis*. The deepest layer consists of the three *plantar interossei*. All the plantar intrinsic muscles are innervated by the medial and lateral plantar branches of the tibial nerve [27].

The dorsal intrinsic muscles of the foot can be divided into two layers [26]. The most superficial layer consists of the *extensor hallucis brevis* and *extensor digitorum brevis*. The deep layer consists of the *dorsal interossei* muscles. The *extensor hallucis brevis* and *extensor digitorum brevis* is innervated by the deep fibular nerve while the *dorsal interossei* are innervated by the lateral plantar nerve with the first and second *dorsal interossei* also receiving part of their innervation from the deep fibular nerve [27]. The dorsal intrinsic muscles have rarely been described in the scientific literature and their function in the foot remains largely unknown [28]. Early EMG studies revealed that the recruitment pattern of the *extensor hallucis brevis* and *extensor digitorum brevis* during walking varied significantly between participants, with some participants demonstrating no activation of *extensor digitorum brevis* during gait [29]*.* The *extensor hallucis brevis* and *extensor digitorum brevis* muscles are now widely used in tissue grafts, such as the island flap to cover soft tissue defects in the distal leg and ankle regions [30]. Therefore, very little is known about the specific roles of dorsal intrinsic muscles and will not be further discussed in this review.

### Evolution of the intrinsic foot muscles

It has been hypothesised that during human evolution, toe flexor force and function are gradually diminishing and therefore plantar intrinsic muscles are becoming largely redundant in the foot [31]. In simian primates, toes are longer and have specialised functions, with toes used to climb trees [32]. Conversely humans have shorter phalanges, which may be a morphological adaptation to the reduced prehensile use of toes in shod wearing modern humans [31]. This theory of adaptive changes during human evolution is supported by the findings of a 3.6 million year old partial human foot, where the toes were shorter than the African ape but longer and more curved than the modern human foot [33]. Some authors have suggested that continued function of some intrinsic muscles may reflect incomplete evolutionary processes [12]. However, the existence of muscles like the *quadratus plantae* disproves this hypothesis. The medial and lateral attachment sites of the *quadratus plantae* muscle into the calcaneus is unique to humans [34] and *quadratus plantae* is unique to the foot as there is no analogous muscle in the hand [34]*.* Since the *flexor digitorum longus* tendon enters the foot from the medial side and pulls the toes medially [35], one theory suggests that the concurrent contraction of the *quadratus plantae* allows the toes to flex in the sagittal plane by redirecting the pull of the *flexor digitorum longus.* This is a necessary development for bipedal ambulation [35]. Therefore, the existence of specialised functions for intrinsic muscles, may suggest that intrinsic foot muscles continue to have a role in the modern foot.

### Role of intrinsic foot muscles

#### Walking

A number of studies reveal that intrinsic foot muscles are active as a group during walking [3,4,36]. A classic electromyography (EMG) study of 12 participants showed that *abductor digiti minimi, abductor hallucis, flexor digitorum brevis*, *dorsal interossei and lumbrical* muscles were all active during the stance phase of gait and continued until toe off [3]. A study by Jacob 2001 combined anthropometrical and plantar pressure data to reveal that *flexor hallucis brevis* (in combination with abductor hallucis) and *flexor digitorum brevis* muscles are able to exert forces approximately 36% and 13% of body weight during the propulsive phase of walking [37]. However, it is unknown whether these muscles act concentrically or eccentrically [31] or have other actions including toe abduction [38]. Mann and Inman [3] suggested that the role of the intrinsic foot muscles is stabilisation of the foot during propulsion. Intrinsic muscle activity during the propulsion phase of gait coincides with passive metatarsophalangeal (MTP) joint dorsiflexion, as the centre of mass moves anterior to the metatarsophalangeal joint. Rolian *et al.*[31] and Goldmann and Bruggemann [39] postulated that the role of the intrinsic and extrinsic toe flexor muscles is to counterbalance the dorsiflexion moment of the ground reaction force at the metatarsophalangeal joint, in the push off phase of walking. This may be achieved by eccentric contraction of the long and short toe flexor muscles to control dorsiflexion at the MTP joint and maintain interphalangeal joint extension, to enable flat toes on the ground until toe off [32,40]. Therefore, by increasing the surface area in contact with the ground, this would improve pressure distribution under the metatarsal heads during walking.

### Arch support

The role of intrinsic muscles in the support of the medial longitudinal arch has been investigated in both standing [3-5] and walking [4,9]. Early EMG studies revealed that intrinsic foot muscles are not active during standing [3-5] and the plantar aponeurosis was widely accepted to be the primary structure responsible for arch support during rest [3,28,35,38]. However, a recent EMG study revealed a small amount of activity in *abductor hallucis, flexor digitorum brevis* and the *quadratus plantae* muscles during relaxed standing with a significant increase in activity with increased postural demands [41]. Reeser *et al.*[35] suggested that intrinsic foot muscles act as trusses for the longitudinal arches, to actively resist bending stresses during walking. This hypothesis is supported by the findings that the plantar aponeurosis tension drops significantly during late stance, while the arch height is increasing [42]. The lack of tension during late stance suggests that other structures such as intrinsic foot muscles may contribute to arch support during propulsion. Furthermore a virtual study of the foot using the Finite Element Method has shown that mechanical stresses on the medial and lateral arch can be adjusted by plantar intrinsic muscles [43]. Therefore there is evidence that intrinsic muscles play an important role in the support of the medial longitudinal arch during gait and a small role in relaxed standing.

### Implications of intrinsic foot muscle weakness

The next section will review the influence of intrinsic muscle weakness in the development of pes cavus in Charcot-Marie-Tooth disease, lesser toe deformities, hallux valgus and heel pain.

### Charcot-Marie-Tooth disease

Charcot-Marie-Tooth disease (CMT) is a peripheral neuropathy, where anatomically distal muscles including the intrinsic muscles are preferentially affected [7]. Weakness of intrinsic foot muscles is a widely accepted pathological finding of CMT and Magnetic Resonance Imaging (MRI) studies have indicated significant atrophy in intrinsic foot muscles [6,7]. Several authors have hypothesised that intrinsic muscle weakness is an important contributor to the development of pes cavus deformity [44][44,45]. One theory suggests that intrinsic muscle atrophy causes dorsiflexion of the MTP joints, due to the unopposed pull of the long extensors of the toe [44]. Dorsiflexion at the MTP joints elevates the longitudinal arch by the windlass effect [44]. The continued imbalance leads to contracture in the plantar fascia and intrinsic muscles, which then pulls the forefoot into plantar flexion, leading to a progressively rigid cavus foot [44,45]. However, a clear causal relationship between intrinsic muscle weakness and the development of pes cavus foot has not been established and other theories of aetiology exist, such as extrinsic invertor-to-evertor muscle imbalance [7]. Without accurate means of evaluating intrinsic muscle strength, the role of intrinsic muscle atrophy in the development of pes cavus deformity will remain unknown.

### Lesser toe deformities

Muscle imbalances between the intrinsic and extrinsic foot muscles have been proposed as the possible cause for lesser toe deformity [10,11,46]. Claw toe deformity is characterised by extension at the MTP joint with flexion of the proximal and distal interphalangeal joints [10]. Hammer toe is characterised by an extended MTP joint, flexed proximal interphalangeal joint and normal or extended distal interphalangeal joint [10]. Claw and hammer toe deformities are common in patients with diabetic neuropathy [47].

In an unaffected foot, the strong extension forces at the MTP joint by the *extensor digitorum longus and brevis* are balanced by the flexors forces produced by long and short toe flexors [10]. However, intrinsic muscle atrophy results in an imbalance of the extensor forces at the MTP joint, leading to the development of toe deformity [10]. The findings of Kwon *et al.*[11] support this theory, where participants with hammer toe deformity had greater disparity in the ratio of toe extensor-to-toe flexor muscle strength compared to the unaffected participants. However, other mechanisms for the development of toe deformity have also been suggested such as restrictive footwear [10,46], rupture of plantar aponeurosis and joint capsule [10]. These alternative theories are supported by the findings of Bus and colleagues [20] in participants with diabetic neuropathy, whereby no difference in the degree of muscle atrophy was found in patients with and without claw deformity. However, a pilot study by Ledoux *et al.*[48] reported that both intrinsic muscle atrophy and increased plantar aponeurosis thickness were present in participants with claw toe deformity. Therefore, multiple factors may contribute to foot and toe deformity. Future prospective studies, measuring intrinsic muscle strength and plantar aponeurosis thickness, may help clarify this relationship.

### Hallux valgus

Hallux valgus, or *bunion*, describes a foot deformity characterised by lateral deviation of the great toe at the MTP joint away from the midline of the body [46]. One proposed cause of hallux valgus deformity is a strength imbalance of the *abductor hallucis* compared to the *adductor hallucis transverse* and *adductor hallucis oblique*[12,13]. When the abductor muscles are weak, it has been suggested that the adductor force becomes dominant, pulling the great toe laterally at the MTP joint [12]. This theory is supported by muscle biopsy findings which revealed histological abnormalities and muscle fibre atrophy in the *abductor hallucis* muscle in patients with symptomatic hallux valgus deformity [13]. Further studies, assessing muscle strength of the individual intrinsic muscles, are needed to better understand the pathogenesis of hallux valgus.

### Heel pain

The role of the intrinsic muscle weakness in the development of plantar heel pain, or plantar fasciitis, is unclear. One theory proposed by Allen and Gross [1] describes a relationship whereby weak intrinsic muscles provide insufficient dynamic truss support to the medial longitudinal arch, causing increased strain on the plantar aponeurosis. A MRI study by Chang *et al.*[8] of participants with chronic unilateral plantar fasciitis, reported a reduction of intrinsic muscle cross-sectional area in the forefoot of the symptomatic foot in comparison to the pain-free foot. The selected reduction of intrinsic foot muscle cross-sectional area in the forefoot and not the rearfoot is interesting because many intrinsic muscles have attachments into the first ray [8]. The atrophy of the intrinsic muscles may affect the stability of the medial longitudinal arch and therefore impede the healing process by further stressing the plantar aponeurosis [8]. Hence, intrinsic muscle weakness may play a significant role in chronic heel pain. However, further research, measuring intrinsic muscle strength prospectively, is needed to confirm this hypothesis.

### Measurement of intrinsic foot muscle strength

The next section will review the ‘direct’ and ‘indirect’ methods of measuring intrinsic muscle strength. The subheading ‘direct methods of assessing intrinsic/extrinsic muscle strength’ reviews the methods that can directly measure a unit of force or power. However, these ‘direct’ methods actually measure toe flexion strength which is a combination of intrinsic and extrinsic muscle strength. The subheading ‘indirect methods of assessing intrinsic muscle strength’ reviews methods that are unable to directly measure force but provide information regarding intrinsic muscle structure and activity.

### Direct methods of assessing intrinsic/extrinsic muscle strength

The direct methods reported in the literature include a variety of clinical tests [11,14,18,19,49,50] and laboratory based tests [15-17]. It is clear that the direct methods reported in the literature primarily measure toe flexor muscle force, while other actions such as toe extension and abduction force are rarely measured. Since toe flexor strength is a combination of intrinsic and extrinsic muscle activity all ‘direct’ methods are actually measuring intrinsic and extrinsic toe muscle strength. A variety of methods have been described that purport to measure toe flexor force: toe hand-held dynamometry; paper grip test; plantar pressure, and the Intrinsic Positive Test [40].

#### Toe dynamometry

Toe dynamometry is an objective tool used to measure toe flexor strength. Different methods of using toe dynamometry have been reported including hand-held dynamometry [50], fixed dynamometry [18], cuff-based fixed dynamometry [11] and a modified hand grip strength tester [19]. The ‘make’ technique was used in all studies whereby the dynamometer is held stationary by an examiner or an external attachment and the participants maximally push down onto the dynamometer with their toes [11,18,19,50]. The reliability of all methods except fixed dynamometry has been reported (Tables 6 and 7). Toe dynamometry has consistently demonstrated excellent intra-rater reliability, with all ICC values > 0.83 [11,19,50]. However, inter-rater reliability has only been reported with hand-held dynamometry, which has shown excellent inter-rater reliability (ICC 0.82 - 0.88) [50].

**Table 6:** Reliability of toe dynamometry

**Method**	**Test**	**Paper**	**Participant**	**Reliability**								**Comment**
				**Statistic**	**Intrarater**			**Interrater**		**Test-retest**		
					**Hallux toe**	**Lesser toes**	**Comb**	**Hallux toe**	**Lesser toes**	**Hallux toe**	**Lesser toe**	
Direct	Toe Dynamometry	Unger & Wooden (2000) [19]	Healthy participants	ICC	0.981	n/a	n/a	n/a	n/a	n/a	n/a	Excellent
			Age 21.0- 62.0 year Sex M & F n=15									
		Kwon *et al.* (2009) [11]	Hammer toe(HT) deformity Vs Matched control	ICC	n/a	n/a	0.88-0.96	n/a	n/a	n/a	n/a	Excellent
			Age: HT 32.0 ± 14.0 year									
			Control 29.0 ± 8.0 year Sex M & F n=29									
		Senda *et al.* (1999) [18]	Marathon Runner Vs participants not involved in sport	n/a	n/a	n/a	n/a	n/a	n/a	n/a	n/a	Not reported
			Age 19.9 ± 1.8 year Sex F n=49									

Legend: Abbreviations: M-Male, F-Female, Comb-combined. Reliability was interpreted in terms of benchmarks suggested by Fleiss [51] where an ICC or Kappa value (excellent reliability, >0.75; fair to good reliability, 0.40–0.75; and poor reliability, <0.4.

**Table 7:** Reliability of toe dynamometry and the paper grip test

**Method**	**Test**	**Paper**	**Participant**	**Reliability**								**Comment**
				**Statistic**	**Intrarater**			**Interrater**		**Test-retest**		
					**Hallux toe**	**Lesser toes**	**Comb**	**Hallux toe**	**Lesser toes**	**Hallux toe**	**Lesser toe**	
Direct	Toe Dynamometry	Spink *et al.* (2010) [50]	Young Vs Older participant	ICC	0.94 (95%CI 0.90-0.96	0.83 (95%CI 0.74-0.89)	n/a	0.88 (95%CI 0.81-0.92)	0.82 (95%CI 0.73-0.89)	n/a	n/a	Excellent
			Age: Young 23.2± 4.3year									
			Older 77.1± 5.7year									
			Sex M & F n=72									
		Goldmann & Bruggemann (2012) [39]	Healthy Participants	Pearson correlation coefficient(r)	n/a	n/a	n/a	n/a	n/a	0.91(combined)		Excellent
			Age 27 ± 3year									
			Sex M n=20									
	Paper Grip Test	De Win *et al.* (2002) [14]	Leprosy Vs healthy control	non-weighted kappa	0.56 (95%CI 0.36-0.76)	0.56 (95%CI 0.39-0.74)	n/a	0.87 (95%CI 0.67-1.0)	0.87 (95%CI 0.34-0.87)	n/a	n/a	Excellent
			Age 30.3year									
			Sex M & F n= 43									
												

Legend: Abbreviations: M-Male, F-Female, Comb-combined. Reliability was interpreted in terms of benchmarks suggested by Fleiss [51] where an ICC or Kappa value (excellent reliability, >0.75; fair to good reliability, 0.40–0.75; and poor reliability, <0.4.

The different types of toe dynamometers allow testing of different actions of the toes. The procedure used to measure toe flexor strength with hand-held dynamometry involves the dynamometer being positioned beneath the interphalangeal joint of the hallux to measure either greater toe strength or interphalangeal joints two to five, for lesser toe strength [50]. As such hand-held dynamometry allows flexion at the MTP joints and limits flexion at the interphalangeal joint because the dynamometer is placed under the interphalangeal joints. In contrast, the modified hand grip strength tester has a bar around which the toes can be flexed [19]. Cuff based fixed dynamometry involves placing a leather cuff around the proximal phalanx of the toe to be measured [11,25]. Cuff based dynamometry has been used to measure both toe flexor [11,25] and extensor muscle strength [11], as the cuff placement and the alignment of the dynamometer can be altered. Fixed dynamometry consists of a fixed sensor plate on which the participants press their toes [18,39].

The different types of toe dynamometry may activate intrinsic muscles to varying degrees because each model promotes different actions of the toes. Cuff-based fixed dynamometry, the modified hand grip tester and fixed dynamometry all allow flexion at the MTP joint, but do not provide a way to limit excessive flexion at the interphalangeal joints. A toe curling action can occur during toe flexor testing, an action which is hypothesised to activate the long (extrinsic) toe flexors [49]. Based on the anatomical insertions of intrinsic foot muscles, primarily the *interossei* and *lumbrical* muscles, Garth and Miller [49] postulated that the intrinsic foot muscles contract as a group, to produce flexion at the MTP joint and extension at the interphalangeal joint. This is in contrast to flexion at both the MTP joint and interphalangeal joint which is an action of the long (extrinsic) flexors of the toes [49]. Studies of the intrinsic muscles of the hand reveal that *interossei* and *lumbrical* muscles can be electrically stimulated to produce flexion at the MTP joint and extension at the interphalangeal joint [52]. Based on the similar anatomy of the *interossei* and *lumbrical* in the hand and foot, flexion at the MTP joint and extension interphalangeal joint are likely actions of intrinsic foot muscles. Therefore, hand-held dynamometry might activate intrinsic muscles more effectively than other types of toe dynamometry because it promotes flexion at the MTP joint and extension at the interphalangeal joint.

Another important consideration when measuring intrinsic muscle strength is the position of the ankle. Spink and co-workers [50] hypothesised that by passively holding the ankle in maximum plantarflexion, the extrinsic toe flexors are less likely to influence the measurement, because these muscles would be in a maximally shortened position and therefore less able to generate force. This hypothesis is supported by the findings of Goldmann and Bruggemann [39] who revealed that the lowest moments of force were generated around the metatarsophalangeal joints when the extrinsic toe flexor muscles were in a shortened position during maximal ankle and metatarsophalangeal joint plantar flexion. The authors suggested that the lower moments were because the intrinsic toe flexor muscles, rather than the extrinsic toe flexor muscles were primarily producing the moments around the metatarsophalangeal joint.

#### Paper grip test

The Paper Grip Test involves the participant attempting to hold a standard piece of paper, like a business card, underneath the hallux or lesser toes while the examiner attempts to pull the card away [14,15]. The Paper Grip Test was first used as a screening tool for intrinsic muscle weakness in leprosy [14]. It has since been used as a measure of toe plantar flexor strength in conjunction with a plantar pressure platform, where the participant performs the Paper Grip Test while sitting with their feet on the platform, which is simultaneously recording forces underneath the toes [15]. The Paper Grip Test has demonstrated excellent interrater (ICC 0.87) and moderate intrarater reliability (ICC 0.56) when assessing participants with leprosy and healthy controls (Table 7).

There are a limited number of validation studies of the Paper Grip test as a measure of intrinsic muscle strength. De Win *et al.*[14] conducted a concurrent EMG test during the paper grip test and revealed that both intrinsic and extrinsic muscles of the foot and ankle were active. Ankle plantarflexor muscle activity may be due to the lack of stabilisation as neither manual stabilisation by the examiner nor straps were used to minimise ankle movement during the study [15]. Furthermore participants may have curled their toes to grip the business card, an action which is hypothesised to activate the long extrinsic toe flexors [49]. Therefore, while the Paper Grip Test is repeatable, it has questionable validity as a measure of intrinsic weakness because is likely to be assessing both intrinsic and extrinsic muscle strength.

#### Plantar pressures

Plantar pressure sensors can assess the force beneath the toes. Plantar pressure measurement is generally available in two different forms: (1) in-shoe systems such as Novel Pedar®, TekScan F-Scan®, RS-Scan Insole®, IVB Biofoot ® [53] and; (2) platform systems such as the Novel Emed®, RSScan Footscan® and TekScan Mat Scan® [54]. Plantar pressure instrumentation has been recently utilised to measure toe flexor strength [15-17]. Toe flexor strength was calculated using software that converted the pressure data under the toes into peak force, which was then normalised to body weight to determine toe flexor strength. Toe flexor strength was assessed on the pressure platform using two different actions: (1) directly pushing the toe into the platform [16,17] and; (2) in conjunction with the Paper Grip Test [15]. The test-retest reliability of the plantar pressure platform was excellent in both methods to assess both great toe and lesser toe strength (Table 8).

**Table 8:** Reliability of plantar pressure measurements

**Method**	**Test**	**Paper**	**Participants**	**Reliability**								**Comment**
				**Statistic**	**Intrarater**			**Interrater**		**Test-retest**		
					**Hallux Toe**	**Lesser toes**	**Comb**	**Hallux toe**	**Lesser toes**	**Hallux toe**	**Lesser toe**	
Direct	Plantar Pressure	Mickle *et al.* (2009) [17]	Fallers (F) Vs	n/a	n/a	n/a	n/a	n/a	n/a	n/a	n/a	Not reported
			Non-fallers (NF)									
			Age F 71.6 ± 6.6 year									
			NF 72.1± 6.7 year									
			Sex M & F n=303									

		Menz *et al.* (2006) [15]	Young Vs Older participants	ICC	n/a	n/a	n/a	n/a	n/a	0.88 (95%CI 0.81-0.93)	0.87 (95%CI 0.73-0.92)	Excellent
			Age: Older 74.6 ± 5.7 year Young 40 ± 2.2 year									
			Sex M & F n=80									

		Mickle *et al.* (2008) [16]	Healthy participants	ICC	n/a	n/a	n/a	n/a	n/a	0.93 ± 0.01	0.92 ± 0.08	Excellent
			Age 31.8 ± 8.5 year							No CI	No CI	
			Sex M & F n=6							reported	reported	
												

Legend: Abbreviations: M-Male, F-Female, Comb-Combined. Reliability was interpreted in terms of benchmarks suggested by Fleiss [51] where an ICC or Kappa value (excellent reliability, >0.75; fair to good reliability, 0.40–0.75; and poor reliability, <0.4.

The validity of using plantar pressures to determine intrinsic muscle strength is questionable because the contribution of the extrinsic muscles during the pressure measurement is unknown. Electromyography performed during Paper Grip Test has revealed that some extrinsic toe flexor muscles, particularly the long toe flexors muscles and ankle plantar flexor muscles were active [14]. Therefore the Paper Grip Test, when used in conjunction with plantar pressure measurement, may reflect extrinsic muscle more than intrinsic muscle strength. Therefore, while the plantar pressure platform is a reliable tool, it has not been extensively investigated as a valid measure of intrinsic muscle strength.

#### Intrinsic positive test

The Intrinsic Positive Test is a qualitative test designed to assess intrinsic muscle function of the lesser toes [49]. The test involves the participant extending the great toe while simultaneously attempting to flex the lesser toes at the MTP joint and extend the interphalangeal joints. The strength of the intrinsic muscles is determined by the type of lesser toe flexion pattern demonstrated which includes either: (1) intrinsic positive pattern, which involves flexion at the MTP joint and extension at the interphalangeal joints; (2) intrinsic negative pattern, where the participant is unable to actively flex the MTP joint and extend the interphalangeal joints [49]. Garth and Miller [49] suggested that the intrinsic negative pattern demonstrated intrinsic muscle weakness. However, the level of strength required to perform the intrinsic positive position is unknown. Furthermore the validity and reliability of the intrinsic positive test has not been investigated and this test has not been cited by any other papers to date. Clearly, the Intrinsic Positive Test has not been extensively investigated as a measure of intrinsic muscle strength and additional research is needed to validate this test.

### Indirect methods of assessing intrinsic muscle strength

The indirect methods that will be reviewed are: Magnetic resonance imaging (MRI); computerised tomography (CT); ultrasonography; electromyography (EMG) and muscle biopsy. Indirect methods are generally used to estimate muscle structure (physiological cross sectional area and volume) [6,21-24,55-57], activity (EMG) [3] and histochemical properties [13]. Indirect methods can discriminate between intrinsic and extrinsic muscles, but are unable to directly determine force or strength.

#### Magnetic resonance imaging

Magnetic resonance imaging (MRI) is the method of choice for detecting soft tissue structure and abnormalities [58,59]. It has been widely used to visualise intrinsic muscles because it has high spatial resolution [7,20,21,55,57,60]. The most commonly used MRI parameter for imaging intrinsic muscle is T1-weighted, which allows a superior contrast to discriminate between muscle and fat [59].

MRI has been used in three main ways to assess intrinsic muscle atrophy: (1) qualitative observation of muscle atrophy [6,7]; (2) five-point scale [20,22]; (3) muscle cross-sectional area and volume [55,57,60]. Qualitative assessment of MR images in 60 participants with CMT has revealed some degree of fatty infiltration and intrinsic muscle atrophy in all participants [6]. Bus *et al.*[21] has also visualised intrinsic muscle atrophy in patients with Diabetes Mellitus, using a five point scale where 0 indicates healthy tissue with no atrophy and 4 indicates a foot with almost no muscle tissue visible. This method has been shown to have good reliability (Kappa = 0.94) (Table 9).

**Table 9:** Reliability of MRI 5 point scale and ultrasonography

**Method**	**Test**	**Paper**	**Participant**	**Parameter**	**Reliability**					**Comment**
					**Statistic**	**Intrarater**		**Interrater**		
						**Within session**	**Between session**	**Hallux toe**	**Lesser toes**	
Indirect	MRI (5 point scale)	Bus *et al.* (2006) [21]	Diabetic patient Vs matched control	Intrinsic muscle atrophy under 5^th^ Metatarsal	weighted kappa	n/a	0.94	n/a	n/a	Excellent
			Age DB 56.8 year							
			Control 58.0 year Sex M & F n=28							
	US (High end Philips HDII)	Hing *et al.* (2009) [61]	Healthy Asymptomatic participants	Dorsoplantar thickness of AbdH	ICC	0.97 (95% CI 0.95-0.99)	0.97 (95%CI 0.95-0.98)	n/a	n/a	Excellent
			Age 28.24 ±10.2 year	Medio-lat width of AbdH	ICC	0.96 (95%CI 0.95-0.98)	0.94 (95%CI 0.90-0.96)	n/a	n/a	
			Sex M & F n=30	CSA of AbdH	ICC	0.98 (95%CI 0.96-0.98)	0.79 (95%CI 0.65-0.88)	n/a	n/a	
	US	Jung *et al.* (2011) [25]	FO vs FOSFE in patient with pes planus	CSA of AbdH	ICC	0.97 (95%CI 0.94-0.99)	n/a	n/a	n/a	Excellent
			Age: FO 21.93 ± 2.73 year							
			FOSFE 22.36 ± 2.41 year n=28							

Legend: Abbreviations: US-Ultrasonography, M-Male, F-Female, AbdH-Abductor hallucis, CSA-cross sectional area, FO- Foot orthosis, FOSFE- Combined FO and short foot exercise. Reliability was interpreted in terms of benchmarks suggested by Fleiss [51] where an ICC or Kappa value (excellent reliability, >0.75; fair to good reliability, 0.40–0.75; and poor reliability, <0.4.

The significant limitation of qualitative observations of muscle atrophy and using the five point scale to assess intrinsic muscle atrophy is that conclusions are based on a selected image, which may not be representative of the entire muscle. The MR images of intrinsic foot muscles can be taken in the coronal [6,7], transverse [6,7] and sagittal planes [20,22]. Since most of the major intrinsic foot muscles originate at the calcaneus and insert onto the proximal phalanges [26], they do not directly lie in the transverse or sagittal planes. Therefore, the images selected for evaluation may not be representative of the entire muscle, because the image is of an oblique slice of the muscle.

The total volume of intrinsic foot muscles can be calculated by multiplying the total cross sectional area of the muscle, determined from MRI, by the distance between the sections, which is the gap between each MRI slice [55,57,60]. The total muscle volume may be more representative of muscle because it is based on the total muscle cross sectional area from each MRI slice and not a single image. Furthermore the MR image can be digitised to outline individual muscles in each slice [62]. However, a study on participants with diabetic neuropathy reported that the segmentation of individual intrinsic muscles was not possible, because most of the muscles were not clearly defined due to the marked intrinsic muscle atrophy [22]. With increasing MRI scanning resolution, future studies may be able to investigate the volumes of individual intrinsic foot muscles.

MRI can also be used to estimate physiological cross-sectional area (PCSA) of intrinsic muscles [62]. PCSA have been used in biomechanical models of muscle dynamics such as the Hill-type muscle model to predict muscle force and torque around the ankle [63-65] and knee joint [63], but not the joints of the foot. The muscle models require the input of a number of different parameters including PCSA, elastic properties of tendons and EMG signals, which are integrated numerically to produce an estimate of muscle force [64,65]. The PCSA can be calculated using muscle volume, the fibre pennation angle and the muscle fibre length [62,65,66]. However, Ledoux *et al.*[64] has showed that intrinsic foot muscles have very small pennation angles and would have little influence on the PCSA. Furthermore the fibre length of intrinsic foot muscles have been investigated in cadaver studies [26] and suggest future studies using PCSA and muscle models may allow intrinsic muscle force to be measured.

An important advance in MRI is the fast-cine phase contrast (or dynamic MRI), which allows images to be acquired while the participant performs an action [67]. Dynamic MRI is different from functional MRI, which is used to map brain function using blood-oxygen signals and cerebral blood flow. At present, only the kinematic properties of the foot, primarily the axis of rotation of talocrural and subtalar joints, have been investigated [67]. Also, at this stage, dynamic MRI is performed in a closed unit and the participant must be supine [67]. Therefore, the image acquired cannot represent full weight bearing and only limited actions can be performed inside the MRI unit, such as ankle plantarflexion/dorsiflexion. However, as dynamic MRI technology improves, studies investigating real time imaging of intrinsic muscles during activities like standing and walking may become available. Thus dynamic MRI could lead to a more accurate appreciation of intrinsic muscles during activities and a better understanding of the action of intrinsic muscles in the future.

#### Computerised tomography

Computerised tomography (CT) is an imaging technique that uses ionising radiation to generate three dimensional images of musculoskeletal structures [59]. CT scans have sufficient resolution to discriminate bone and muscles [59] and have been used in many earlier studies to estimate muscle size [2,23,68]. Robertson *et al.*[23] and Mueller *et al.*[24] performed CT scans to assess soft tissue density under the second metatarsal shaft, as a proxy measure of intrinsic muscle size in a patient with diabetic neuropathy. However, both studies reported difficulty defining the borders of intrinsic muscles, which may be related to the insufficient contrast resolution of CT scans, compared with MRI [23]. Furthermore, the main limitation of CT to assess intrinsic muscle size is the exposure to harmful ionising radiation [59].

#### Ultrasonography

Ultrasonography is a technique that uses mechanically produced longitudinal sound waves to create an image [59]. Ultrasonography has been used to measure dimensional parameters of intrinsic foot muscles including cross sectional area [25,55,61,69,70], dorso-plantar thickness [55,61,69,70] and medio-lateral width [61,70]. Ultrasound has been used to investigate intrinsic muscles as a group [55,69] such as the muscles between the first and second metatarsal bone including the first *dorsal interosseus* muscle, *adductor hallucis muscle*, and the first *lumbrical* muscle [55]. More recently individual intrinsic muscles have also been investigated: *abductor hallucis*[25,61,69], *abductor digiti minimi*[69], *flexor hallucis brevis*[69], *quadratus plantae*[69], *extensor digitorum brevis*[55]. Studies using ultrasonography to measure intrinsic muscle parameters have consistently demonstrated excellent intra-rater reliability (Table 9, 10 and 11). A study by Hing *et al.*[61] revealed good-excellent intra-rater reliability (ICC between 0.64-0.97) in both a high end and portable ultrasound unit when used to assess cross sectional area and muscle thickness in *abductor hallucis* muscles.

**Table 10:** Reliability of ultrasonography

**Method**	**Test**	**Paper**	**Participant**	**Parameter**	**Reliability**					**Comment**
					**Statistic**	**Intrarater**		**Interrater**		
						**Within session**	**Between session**	**Hallux toe**	**Lesser toes**	
Indirect	US (Chison 8300 Deluxe Digital System-Portable Model)	Hing *et al.* (2009) [61]	Healthy Asymptomatic participants	Dorsoplantar thickness of AbdH	ICC	0.979(95% CI 0.99-0.99)	0.96 (95%CI 0.93-0.97)			
			Age 28.24 ±10.2 year							
			Sex M & F n=30							
				Medio-lat width of AbdH	ICC	0.95 (95%CI 0.92-0.97)	0.88 (95%CI 0.80-0.93)			
				CSA	ICC	0.99 (95%CI 0.98-0.99)	0.78 (95%CI 0.64-0.87)			
	US (Philips HDII-High end model)	Cameron *et al.* (2008) [70]	Healthy Asymptomatic participants	Dorso-plantar thickness of AbdH	ICC	0.97(95% CI 0.98-0.99)	0.97(95%CI 0.93-0.98)			
			Age 28.24 ± 10.2 year							
			Sex M & F n=30							
				Medio-lat width of AbdH	ICC	0.97(95% CI 0.92-0.97)	0.94 (95%CI 0.90-0.96)			
				CSA	ICC	0.98 (95%CI 0.98-0.979)	0.79 (95%CI 0.65-0.88)			

Legend: Abbreviations: US-Ultrasonography, M-Male, F-Female, AbdH-Abductor hallucis, CSA-cross sectional area. Reliability was interpreted in terms of benchmarks suggested by Fleiss [51] where an ICC or Kappa value (excellent reliability, >0.75; fair to good reliability, 0.40–0.75; and poor reliability, <0.4.

**Table 11:** Reliability of ultrasonography continued

**Method**	**Test**	**Paper**	**Participant**	**Parameter**	**Reliability**					**Comment**
					**Statistic**	**Intrarater**		**Interrater**		
						**Within session**	**Between session**	**Hallux toe**	**Lesser toes**	
Indirect	US	Mickle *et al.* (2012) [69]	Adults	AbdH CSA	ICC	n/a	0.98	n/a	n/a	Excellent
			Age 33.1 ± 11.2 year	AbdH Thickness	ICC	n/a	0.98	n/a	n/a	Excellent
			Sex M & F n=8	FHB CSA	ICC	n/a	0.83	n/a	n/a	Excellent
				FHB Thickness	ICC	n/a	0.94	n/a	n/a	Excellent
				FDB CSA	ICC	n/a	0.99	n/a	n/a	Excellent
				FDB Thickness	ICC	n/a	0.87	n/a	n/a	Excellent
				QP CSA	ICC	n/a	0.99	n/a	n/a	Excellent
				QP Thickness	ICC	n/a	0.95	n/a	n/a	Excellent
				AbdM CSA	ICC	n/a	0.97	n/a	n/a	Excellent
				AbdM Thickness	ICC	n/a	0.96	n/a	n/a	Excellent

Legend: Abbreviations: US-Ultrasonography, M-Male, F-Female, AbdhH-abductor hallucis, FHB-flexor hallucis brevis, FDB- flexor digitorum brevis, QP- quadratus plantae, AbdM-abductor digiti minimi, CSA-cross sectional area. Reliability was interpreted in terms of benchmarks suggested by Fleiss [51] where an ICC or Kappa value (excellent reliability, >0.75; fair to good reliability, 0.40–0.75; and poor reliability, <0.4.

Ultrasonography has two main limitations; low spatial resolution of the image [57], and the quality of the measurement is operator-dependant [59]. The low resolution capabilities of ultrasonography means it cannot identify areas of fatty infiltration in muscles [57] and therefore may over estimate intrinsic muscle size and underestimate intrinsic muscle atrophy. The reliability studies of intrinsic muscle measurement using ultrasonography have assessed between and within session intra-rater reliability. However, only ultrasonography studies on measurement of larger intrinsic muscles of the back have demonstrated excellent inter-rater reliability (ICC between 0.85-0.97) [71]. Therefore further research assessing inter-rater reliability, and comparing to MRI of muscle size is required before ultrasonography can be established as a reliable and valid tool for evaluating intrinsic foot muscle parameters.

#### Electromyography

Electromyography (EMG) has been assessed by non-invasive surface electrodes and invasive intramuscular electrodes [72]. Surface EMG places the electrodes directly on the skin and therefore the signal is a combination of all the muscle fibre action potentials occurring in the muscles underlying the skin electrodes [72]. A surface EMG study by Arinci *et al.*[12] recorded the mean amplitude of the EMG signal, to make inferences concerning the level of intrinsic muscle activity. The relationship between EMG amplitude and the amount of muscle activity is problematic because the EMG signal amplitude predominately consists of action potentials from the muscle fibres closest to the recording tip of the electrode and may not record activity from all the active muscle fibres [73]. Therefore the mean amplitude of the EMG signal may not be an accurate measure of the level of muscle activity or muscle strength.

Intramuscular EMG involves needle electrodes placed directly in the muscle. Most intramuscular EMG studies of intrinsic foot muscles insert needle electrodes into individual muscle bellies [3-5,28]. However, only one recent study confirmed the identity of the muscle using real time ultrasonography [41]. Intramuscular EMG can detect intrinsic muscle activation patterns and therefore provide valuable insight into the function of the intrinsic muscles during a particular task. A recent EMG study assessed the activation patterns and mean EMG signal amplitude in *abductor hallucis, flexor digitorum brevis*, *dorsal interossei* and *quadratus plantae* during standing task with increasing postural difficulty. The study revealed increased EMG signal amplitude in all muscles with increasing postural demands of the task, as assessed by deviation of the centre of pressure [41]. The limitation of using needle electrodes is that they record activity from a smaller number of muscle fibres and therefore may not detect subtle muscle contractions [73]. Therefore EMG can detect individual intrinsic muscle activity, but cannot be used to assess intrinsic muscle strength.

#### Muscle biopsy

Muscle biopsy can be used to detect changes in muscle histology and ultrastructure [74]. Biopsy samples can be stained to assess the relative number, size and distribution of the fibres within the sample and detect atrophied muscle fibres [2,13,74]. Hoffmeyer *et al.*[13] performed muscle biopsies on the *abductor hallucis* muscles in participants with symptomatic hallux valgus and reported histological abnormalities including muscle fibres atrophy, lipid laden fibres and ultrastructural changes in the mitochondria. However, the limitation of muscle biopsies is that findings may not be representative of the whole muscle belly [74]. Therefore, limited inferences can be made regarding whole muscle behaviour and function solely from muscle biopsy.

## Discussion

There is no gold standard method to measure intrinsic muscle strength in the foot. At present methods of measuring intrinsic muscle strength can be categorised as direct and indirect methods. Direct methods are able to quantify [11,15-19,50] or semi-quantify muscle force/strength [14,49]. The main limitation of direct method is that all direct methods have limited specificity and usually assess global toe flexor strength as they are unable to isolate intrinsic muscle from extrinsic muscle strength. Direct methods such as the paper grip test [14] and plantar pressures [15-17], have lower reliability than hand-held dynamometry [50]. In addition, these methods allow the toe curling action to occur during the strength measurement, which is an action associated with the contraction of extrinsic foot muscles [49]. In contrast, hand-held dynamometry has high reliability [50] and allows flexion at the MTP joint and extension at the interphalangeal joint, which is an action hypothesised to activate the foot intrinsic muscles [49]. Therefore, hand-held dynamometry is a promising method of measuring intrinsic muscle strength.

However, hand-held dynamometry has some barriers to accurately measure intrinsic muscle strength. First, it is currently unknown which intrinsic muscles are active during the hand-held dynamometry test. Second, it is unclear what action should be performed by the toes to best activate the individual intrinsic muscles during the dynamometry test. Garth and Miller [49] hypothesised that the intrinsic muscle contraction produces flexion at the MTP joint and extension at the interphalangeal. However, this approach has not been directly evaluated in the foot and has only been inferred from studies in the hand. Furthermore hand-held dynamometry has only been used to measure toe flexor strength and not toe extensor or abductor strength. While the implications of toe extensor and abductor weakness of the foot is unclear, an accurate measure of toe extensor, abductor and adductor strength may provide valuable insight into muscle imbalances in evolving foot deformity [11]. Therefore, an ideal method of measuring intrinsic muscle strength must be able to differentiate the strength of intrinsic extensor, adductor and abductor muscles in the foot. Third, it is unclear whether hand-held dynamometry can actually isolate intrinsic muscles during strength testing. While it is unlikely that hand-held dynamometry measures only intrinsic muscle strength, it is important to know the relative contribution of the intrinsic and extrinsic muscles during the measurement. An EMG study with intra-muscular electrodes inserted using real time ultrasound imaging into both intrinsic and extrinsic muscles during dynamometry testing may provide valuable insight into muscle activation patterns of both intrinsic and extrinsic muscles during toe flexor testing. Therefore, isolated intrinsic muscle contraction must be demonstrated before hand-held dynamometry can be accepted as a valid measure of intrinsic muscle strength.

Indirect methods such as imaging modalities [6,21-24,55-57], electromyography [3,12] and muscle biopsy [13] are able to discriminate between intrinsic and extrinsic muscles. Indirect methods can assess muscle morphology [6,21-24,55-57], activation patterns [3,12,41] and histochemical properties [13] of intrinsic foot muscles. However, indirect methods are unable to directly measure intrinsic muscle strength. Among the imaging modalities, MRI seems to be the most effective method to visualise intrinsic muscles of the foot because of its multiplanar views [58,59], safety [2,58,59] and high resolution [58,59]. In contrast, CT uses ionising radiation [2,58,59] and has lower contrast resolution compared to MRI [58]. Ultrasonography has the lowest resolution of the three imaging modalities [2,59,68] and the reproducibility of the image is operator dependant [59]. Muscle biopsy [13,74] and intramuscular EMG [73] assess only a small number of muscle fibres and thus the findings may not be representative of the whole muscle. Therefore, it seems MRI is the best indirect method to assess intrinsic muscle morphological properties such as PCSA and volume to identify muscle atrophy. A relationship between muscle volume and its force generating capacity in the foot has only been demonstrated in ankle dorsiflexor and plantar flexor muscles. Other factors such as neural drive and biomechanical consideration such as muscle-tendon length and moment arms of muscles may also impact the force generating capacity of muscles [39,75]. Biomechanical muscle modelling based on intrinsic muscle morphological data from MRI represents a promising new approach for quantifying intrinsic muscle force in the future [63,65]. However, one of the main challenges with using mathematical models to predict muscle force depends on the accuracy of its parameters [65] such as muscle fibre length and pennation angle, which are often taken from cadaver studies. At present, muscle modelling has only been used to accurately predict muscle forces and torques around the ankle [65] and knee joints [63].

### Future research direction

This review suggests that at present, there is no adequately validated method of measuring intrinsic muscle strength. The main challenges are that no direct method can isolate intrinsic muscle strength from extrinsic muscle strength, and indirect methods cannot quantify muscle force. Future research in this field would benefit from using a combination of indirect and direct methods to measure intrinsic muscle force, because both measures have their strengths and limitations. For instance, a study comparing muscle architectural information from MRI, muscle modelling estimates of muscle force and muscle activation data from EMG, with the findings of direct methods such as hand held dynamometry would disentangle each method’s limitation. Also indwelling EMG during hand-held dynamometry would enable both intrinsic and extrinsic muscle activity patterns to be compared, although this approach has numerous practical and analytical considerations to overcome. Nevertheless, a strong correlation would validate the findings of direct methods used to measure intrinsic muscle strength.

An accurate and reliable measure of intrinsic muscle strength will enable prospective studies to address the causal relationship questions between intrinsic muscle weakness and foot/toe deformity in conditions like Charcot-Marie-Tooth disease [6,7], diabetic neuropathy [10,11,20], hallux valgus [10,12,13] and heel pain [1,8,9]. Furthermore a validated method of measuring intrinsic muscle weakness can guide future research into clinical trials of intrinsic strength training in a variety of clinical populations. Overall, a better measurement of intrinsic muscle strength is necessary to improve the clinical management of these conditions.

## Conclusion

In conclusion, measuring intrinsic muscle strength poses many challenges. Most studies have focussed on toe flexor strength as a surrogate measure of intrinsic muscle strength, while other actions of toe muscles have not been assessed. Hand-held dynamometry represents a promising method of estimating intrinsic muscle strength. However, the contribution of extrinsic muscles cannot be excluded from toe flexor strength measurement. Future research should clarify the relative contribution of intrinsic and extrinsic muscles during intrinsic foot muscle strength testing.

## Competing interests

The authors declare that they have no competing interests.

## Authors’ contributions

AS searched the literature, analysed the articles and drafted the review. JB, CH and KR conceptualised and edited the review. All authors read and approved the final manuscript.

## References

[B1] Allen RH Gross MT Toe flexors strength and passive extension range of motion of the first metatarsophalangeal joint in individuals with plantar fasciitis J Orthop Sports Phys Ther 2003 33 468 478 12968860 10.2519/jospt.2003.33.8.468

[B2] Alanen AM Falck B Kalimo H Komu ME Sonninen VH Ultrasound, computed tomography and magnetic resonance imaging in myopathies: correlations with electromyography and histopathology Acta Neurol Scand 1994 89 336 346 8085431 10.1111/j.1600-0404.1994.tb02644.x

[B3] Mann R Inman VT Phasic activity of intrinsic muscles of the foot J Bone Joint Surg Am 1964 46 469 481 14131426

[B4] Basmajian JV Stecko G The role of muscles in arch support of the foot: electromyographic study J Bone Joint Surg Am 1963 45 1184 1190 14077983

[B5] Basmajian J Bentzon J An electromyographic study of certain muscles of the foot in the standing position Surg Gynecol Obstet 1954 98 662 666 13168829

[B6] Chung KW Suh BC Shy ME Cho SY Yoo JH Park SW Moon H Park KD Choi KG Kim S Different clinical and magnetic resonance imaging features between Charcot-Marie-Tooth disease type 1A and 2A Neuromuscul Disord 2008 18 610 618 10.1016/j.nmd.2008.05.012 18602827

[B7] Gallardo E García A Combarros A Berciano J Charcot–Marie–Tooth disease type 1A duplication: spectrum of clinical and magnetic resonance imaging features in leg and foot muscles Brain 2006 129 426 437 16317020 10.1093/brain/awh693

[B8] Chang R Kent-Braun J Van Emmerik E Hamill J Distribution of Intrinsic Foot Muscles in Healthy and Plantar Fasciitis Feet2nd Congress of the International Foot and Ankle Biomechanics Community 2010 Seattle, USA: Journal of Foot and Ankle Research

[B9] Headlee D Leonardb J Harta J Ingersolla C Hertela J Fatigue of the plantar intrinsic foot muscles increases navicular drop J Electromyogr Kinesiol 2008 18 420 425 10.1016/j.jelekin.2006.11.004 17208458

[B10] Myerson MS Shereff MJ The pathological anatomy of claw and hammer toes J Bone Joint Surg Am 1989 71 45 49 2913002

[B11] Kwon OY Tuttle LJ Johnson JE Mueller MJ Muscle imbalance and reduced ankle joint motion in people with hammer toe deformity Clin Biomech (Bristol, Avon) 2009 24 670 675 10.1016/j.clinbiomech.2009.05.010 PMC2751588 19535185

[B12] Arinci Incel N Genc H Erdem HR Yorgancioglu ZR Muscle imbalance in hallux valgus: an electromyographic study Am J Phys Med Rehabil 2003 82 345 349 12704272 10.1097/01.PHM.0000064718.24109.26

[B13] Hoffmeyer P Cox JN Blanc Y Meyer JM Taillard W Muscle in hallux valgus Clin Orthop Relat Res 1988 232 112 118 3383479

[B14] de Win MM Theuvenet WJ Roche PW de Bie RA van Mameren H The paper grip test for screening on intrinsic muscle paralysis in the foot of leprosy patients Int J Lepr Other Mycobact Dis 2002 70 16 24 12120036

[B15] Menz HB Zammit GV Munteanu SE Genevieve S Plantarflexion strength of the toes: age and gender differences and evaluation of a clinical screening test Foot Ankle Int 2006 27 1103 1108 17207439 10.1177/107110070602701217

[B16] Mickle KJ Chambers S Steele JR Munro BJ A novel and reliable method to measure toe flexor strength[abstract] Clin Biomech (Bristol, Avon) 2008 23 683 683 10.1016/j.clinbiomech.2008.03.025

[B17] Mickle KJ Munro BJ Lord SR Menz HB Steele JR ISB Clinical Biomechanics Award 2009: toe weakness and deformity increase the risk of falls in older people Clin Biomech (Bristol, Avon) 2009 24 787 791 10.1016/j.clinbiomech.2009.08.011 19751956

[B18] Senda M Takahara Y Yagata Y Yamamoto K Nagashima H Tukiyama H Inoue H Measurement of the muscle power of the toes in female marathon runners using a toe dynamometer Acta Med Okayama 1999 53 189 191 10488406 10.18926/AMO/31617

[B19] Unger CL Wooden MJ Effect of foot intrinsic muscle strength training on jump performance. [article] J Strength Cond Res 2000 14 373 378

[B20] Bus S Maas M Michels P Levi M Role of Intrinsic Muscle Atrophy in the Etiology of Claw Toe Deformity in Diabetic Neuropathy May Not Be as Straightforward as Widely Believed Diabetes Care 2009 32 1063 1067 10.2337/dc08-2174 19279305 PMC2681028

[B21] Bus SA Maas M Lindeboom R Reproducibility of foot structure measurements in neuropathic diabetic patients using magnetic resonance imaging J Magn Reson Imaging 2006 24 25 32 10.1002/jmri.20601 16736473

[B22] Bus SA Yang QX Wang JH Smith MB Wunderlich R Cavanagh PR Intrinsic muscle atrophy and toe deformity in the diabetic neuropathic foot: a magnetic resonance imaging study Diabetes Care 2002 25 1444 1450 10.2337/diacare.25.8.1444 12145248

[B23] Robertson DD Mueller MJ Smith KE Commean PK Pilgram T Johnson JE Structural changes in the forefoot of individuals with diabetes and a prior plantar ulcer J Bone Joint Surg Am 2002 84 1395 1404 12177270 10.2106/00004623-200208000-00016

[B24] Mueller MJ Smith KE Commean PK Robertson DD Johnson JE Use of computed tomography and plantar pressure measurement for management of neuropathic ulcers in patients with diabetes Phys Ther 1999 79 296 307 10078773

[B25] Jung D-Y Koh E-K Kwon O-Y Effect of foot orthoses and short-foot exercise on the cross-sectional area of the abductor hallucis muscle in subjects with pes planus: A randomized controlled trial J Back Musculoskelet Rehabil 2011 24 225 231 22142711 10.3233/BMR-2011-0299

[B26] Kura H Luo ZP Kitaoka HB An KN Quantitative analysis of the intrinsic muscles of the foot Anat Rec 1997 249 143 151 10.1002/(SICI)1097-0185(199709)249:1<143::AID-AR17>3.0.CO;2-P 9294659

[B27] Gray H Gray’s anatomy 1989 37 Edinburgh: C. Livingstone

[B28] Basmajian J DeLuca C: Muscles alive 1985 Their functions revealed by electromyography. Baltimore: Williams and Wilkins

[B29] Cd C König BJ Vitti M Electromyographic study of the muscles "extensor digitorum brevis" and "extensor hallucis brevis" Rev Hosp Clin Fac Med Sao Paulo 1967 22 65 72 5602194

[B30] Baltensperger MM Ganzoni N Jirecek V Meyer VE The extensor digitorum brevis island flap: possible applications based on anatomy Plast Reconstr Surg 1998 101 107 113 10.1097/00006534-199801000-00018 9427923

[B31] Rolian C Lieberman DE Hamill J Scott JW Werbel W Walking, running and the evolution of short toes in humans J Exp Biol 2009 212 713 721 10.1242/jeb.019885 19218523

[B32] Mann RA Hagy JL The function of the toes in walking, jogging and running Clin Orthop Relat Res 1979 142 24 29 498642

[B33] Latimer BM Lovejoy CO Johanson DC Coppens Y Hominid tarsal, metatarsal, and phalangeal bones recovered from the Hadar formation: 1974–1977 collections Am J Phys Anthropol 1982 57 701 719 10.1002/ajpa.1330570412

[B34] Lewis OJ The comparative morphology of M. flexor accessorius and the associated long flexor tendons J Anat 1962 96 321 333 14464897 PMC1244117

[B35] Reeser L Susman R Stern J Electromyographic studies of the human foot: experimental approaches to hominid evolution Foot Ankle 1983 3 391 407 6409717 10.1177/107110078300300607

[B36] Chang R Larsen R Kent-Braun J Hamill J Energetics of the intrinsic foot muscles in plantar fasciitis1st Congress of the International Foot & Ankle Biomechanics (i-FAB) community 2008 Bologna: Journal of Foot and Ankle Research

[B37] Jacob HAC Forces acting in the forefoot during normal gait - an estimate Clin Biomech (Bristol, Avon) 2001 16 783 792 10.1016/S0268-0033(01)00070-5 11714556

[B38] Wong YS Influence of the abductor hallucis muscle on the medial arch of the foot: a kinematic and anatomical cadaver study Foot Ankle Int 2007 28 617 620 10.3113/FAI.2007.0617 17559771

[B39] Goldmann J-P Brüggemann G-P The potential of human toe flexor muscles to produce force J Anat 2012 221 187 194 10.1111/j.1469-7580.2012.01524.x 22747582 PMC3406365

[B40] Nihal A Goldstein J Haas J Hiebert R Kummer FJ Liederbach M Trepman E Toe flexor forces in dancers and non-dancers Foot Ankle Int 2002 23 1119 1123 12503803 10.1177/107110070202301207

[B41] Kelly LA Kuitunen S Racinais S Cresswell AG Recruitment of the plantar intrinsic foot muscles with increasing postural demand Clin Biomech (Bristol, Avon) 2012 27 46 51 10.1016/j.clinbiomech.2011.07.013 21864955

[B42] Caravaggi P Pataky T Günther M Savage R Crompton R Dynamics of longitudinal arch support in relation to walking speed: contribution of the plantar aponeurosis J Anat 2010 217 254 261 10.1111/j.1469-7580.2010.01261.x 20646107 PMC2972539

[B43] Wu L Nonlinear finite element analysis for musculoskeletal biomechanics of medial and lateral plantar longitudinal arch of Virtual Chinese Human after plantar ligamentous structure failures Clin Biomech (Bristol, Avon) 2007 22 221 229 10.1016/j.clinbiomech.2006.09.009 17118500

[B44] Sabir M Lyttle D Pathogenesis of pes cavus in Charcot-Marie-Tooth disease Clin Orthop Relat Res 1983 175 173 178 6839584

[B45] Mann D Hsu J Triple arthrodesis in the treatment of fixed cavovarus deformity in adolescent patients with Charcot-Marie-Tooth disease Foot Ankle 1992 13 1 1 6 1577335 10.1177/107110079201300101

[B46] Coughlin M Mallet toes, hammer toes, claw toes, and corns: causes and treatment of lesser-toe deformities Postgrad Med 1984 75 191 198 6709528 10.1080/00325481.1984.11698001

[B47] Smith D Barnes B Sands A Boyko E Ahroni J Prevalence of radiographic foot abnormalities in patients with diabetes Foot Ankle Int 1997 18 342 346 9208292 10.1177/107110079701800606

[B48] Ledoux W Schoen J Lovell M Huff E Clawed toes in the diabetic foot: neuropathy, intrinsic muscle volume, and plantar aponeurosis thickness[abstract] J Foot Ankle Res 2008 1 1 2 10.1186/1757-1146-1-1 18822156 PMC2547890

[B49] Garth WP Jr Miller ST: Evaluation of claw toe deformity, weakness of the foot intrinsics, and posteromedial shin pain Am J Sports Med 1989 17 821 827 10.1177/036354658901700617 2624294

[B50] Spink M Fotoohabadia M Menz H Foot and Ankle Strength Assessment Using Hand-Held Dynamometry: Reliability and Age-Related Differences Gerontology 2010 56 6 525 532 10.1159/000264655 19955706

[B51] Fleiss JL Reliability of Measurement The design and analysis of clinical experiments 1986 New York: Wiley

[B52] Lauer RT Kilgore KL Peckham PH Bhadra N Keith MW The function of the finger intrinsic muscles in response to electrical stimulation IEEE Trans Rehabil Eng 1999 7 19 26 10.1109/86.750547 10188604

[B53] Zammit GV Menz HB Munteanu SE Reliability of the TekScan MatScan® system for the measurement of plantar forces and pressures during barefoot level walking in healthy adults J Foot Ankle Res 2010 3 11 10.1186/1757-1146-3-11 20565812 PMC2902454

[B54] Orlin M McPoil T Plantar pressure assessment Phys Ther 2000 80 399 409 10758524 10.1093/ptj/80.4.399

[B55] Severinsen K Obel A Jakobsen J Andersen H Atrophy of Foot Muscles in Diabetic Patients Can Be Detected With Ultrasonography Diabetes Care 2007 30 3053 3057 10.2337/dc07-0108 17717286

[B56] Brash PD Foster J Vennart W Anthony P Tooke JE Magnetic resonance imaging techniques demonstrate soft tissue damage in the diabetic foot Diabet Med 1999 16 55 61 10.1046/j.1464-5491.1999.00005.x 10229294

[B57] Severinsen K Andersen H Evaluation of atrophy of foot muscles in diabetic neuropathy - A comparative study of nerve conduction studies and ultrasonography Clin Neurophysiol 2007 118 2172 2175 10.1016/j.clinph.2007.06.019 17709290

[B58] Flemming DJ Murphey MD McCarthy K Imaging of the foot and ankle: Summary and update Curr Opin Orthop 2005 16 54 59 10.1097/01.bco.0000154176.29585.8b

[B59] Kuo GP Carrino JA Skeletal muscle imaging and inflammatory myopathies Curr Opin Rheumatol 2007 19 530 535 10.1097/BOR.0b013e3282efdc66 17917531

[B60] Andersen H Gjerstad MD Jakobsen J Atrophy of foot muscles: A measure of diabetic neuropathy Diabetes Care 2004 27 2382 2385 10.2337/diacare.27.10.2382 15451904

[B61] Hing W Rome K Cameron A Reliability of measuring abductor hallucis muscle parameters using two different diagnostic ultrasound machines J Foot Ankle Res 2009 2 33 10.1186/1757-1146-2-33 19917127 PMC2784754

[B62] Fukunaga T Roy RR Shellock FG Hodgson JA Day MK Lee PL Kwong-Fu H Edgerton VR Physiological cross-sectional area of human leg muscles based on magnetic resonance imaging J Orthop Res 1992 10 928 934 1403308 10.1002/jor.1100100623

[B63] Lloyd DG Besier TF An EMG-driven musculoskeletal model to estimate muscle forces and knee joint moments in vivo J Biomech 2003 36 765 776 10.1016/S0021-9290(03)00010-1 12742444

[B64] Ledoux WR Hirsch BE Church T Caunin M Pennation angles of the intrinsic muscles of the foot J Biomech 2001 34 399 403 10.1016/S0021-9290(00)00194-9 11182133

[B65] de Oliveira LF Luporini Menegaldo L Individual-specific muscle maximum force estimation using ultrasound for ankle joint torque prediction using an EMG-driven Hill-type model J Biomech 2010 43 2816 2821 10.1016/j.jbiomech.2010.05.035 20541763

[B66] Ward S Eng C Smallwood L Lieber R Are current measurements of lower extremity muscle architecture accurate? Clin Orthop Relat Res 2009 467 1074 1082 10.1007/s11999-008-0594-8 18972175 PMC2650051

[B67] Sheehan F The instantaneous helical axis of the subtalar and talocrural joints: a non-invasive in vivo dynamic study J Foot Ankle Res 2010 3 13 10.1186/1757-1146-3-13 20626876 PMC2912255

[B68] Schedel H Reimers CD Witt TN Pongratz DE Vogl T x00E Imaging techniques in myotonic dystrophy. A comparative study of ultrasound, computed tomography and magnetic resonance imaging of skeletal muscles Eur J Radiol 1992 15 230 238 10.1016/0720-048X(92)90113-N 1490449

[B69] Mickle K Nester C Crofts G Steele J Reliability of ultrasound to measure morphology of the toe flexor muscles[abstract] J Foot Ankle Res 2012 5 1 2 10.1186/1757-1146-5-1 23557252 PMC3621612

[B70] Cameron A Rome K Hing W Ultrasound evaluation of the abductor hallucis muscle: reliability study J Foot Ankle Res 2008 1 12 10.1186/1757-1146-1-12 18822116 PMC2565658

[B71] Wallwork T Hides J Stanton W Intrarater and interrater reliability of assessment of lumbar multifidus thickness using rehabilitative ultrasound imaging J Orthop Sports Phys Ther 2007 37 608 612 17970407 10.2519/jospt.2007.2418

[B72] Reaz M Hussain M Mohd-Yasin F Techniques of EMG signal analysis: detection, processing, classification and applications (Correction) Biol Proced Online 2006 8 163 163 10.1251/bpo124 19565309 PMC1622762

[B73] Daube JR Rubin DI Needle electromyography Muscle Nerve 2009 39 244 270 10.1002/mus.21180 19145648

[B74] Dubowitz V, Sewry C Muscle biopsy: a practical approach 2007 3 Philadelphia: Saunders/Elsevier

[B75] Gadeberg P Andersen H Jakobsen J Volume of ankle dorsiflexors and plantar flexors determined with stereological techniques J Appl Physiol 1999 86 1670 1675 10.1063/1.370945 10233134

